# Evidence for charge delocalization crossover in the quantum critical superconductor CeRhIn_5_

**DOI:** 10.1038/s41467-023-42965-1

**Published:** 2023-11-13

**Authors:** Honghong Wang, Tae Beom Park, Jihyun Kim, Harim Jang, Eric D. Bauer, Joe D. Thompson, Tuson Park

**Affiliations:** 1 Center for Quantum Materials and Superconductivity (CQMS), Sungkyunkwan University, Suwon, South Korea; 2 Department of Physics, Sungkyunkwan University, Suwon, South Korea; 3 Institute of Basic Science, Sungkyunkwan University, Suwon, South Korea; 4 Los Alamos National Laboratory, Los Alamos, NM USA

**Keywords:** Phase transitions and critical phenomena, Superconducting properties and materials

## Abstract

The nature of charge degrees-of-freedom distinguishes scenarios for interpreting the character of a second order magnetic transition at zero temperature, that is, a magnetic quantum critical point (QCP). Heavy-fermion systems are prototypes of this paradigm, and in those, the relevant question is where, relative to a magnetic QCP, does the Kondo effect delocalize their *f*-electron degrees-of-freedom. Herein, we use pressure-dependent Hall measurements to identify a finite-temperature scale *E*_loc_ that signals a crossover from *f*-localized to *f*-delocalized character. As a function of pressure, *E*_loc_(*P*) extrapolates smoothly to zero temperature at the antiferromagnetic QCP of CeRhIn_5_ where its Fermi surface reconstructs, hallmarks of Kondo-breakdown criticality that generates critical magnetic and charge fluctuations. In 4.4% Sn-doped CeRhIn_5_, however, *E*_loc_(*P*) extrapolates into its magnetically ordered phase and is decoupled from the pressure-induced magnetic QCP, which implies a spin-density-wave (SDW) type of criticality that produces only critical fluctuations of the SDW order parameter. Our results demonstrate the importance of experimentally determining *E*_loc_ to characterize quantum criticality and the associated consequences for understanding the pairing mechanism of superconductivity that reaches a maximum *T*_c_ in both materials at their respective magnetic QCP.

## Abstract (web summary)

Heavy-fermion superconductors feature a magnetic quantum critical point linked to the Kondo effect breakdown. Wang et al. use pressure-dependent Hall measurements to identify a crossover energy scale, confirming this in pure CeRhIn_5_, while revealing a shift to spin density wave criticality with Sn-doping.

## Introduction

**Fig. 1:**
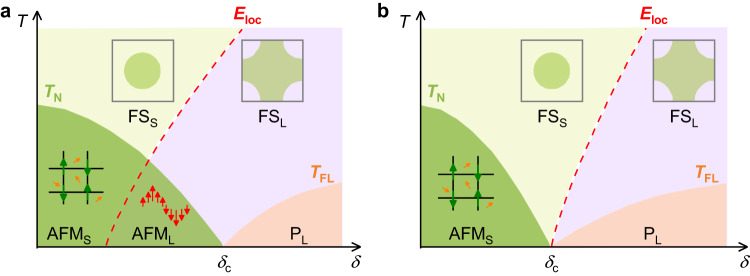
Schematic phase diagrams of two classes of QCPs in AFM heavy-fermion metals. Under the variation of the non-thermal parameter *δ*, an AFM transition is continuously suppressed to zero temperature at a critical value *δ*_c_. Consequently, an AFM QCP appears at *δ*_c_ and the ground state changes from an AFM to a paramagnetic Fermi-liquid (FL) state. *T*_N_ and *T*_FL_ represent the AFM transition temperature and the onset temperature of the FL regime, respectively. *E*_loc_ denotes the Kondo destruction energy scale that is associated with delocalization of the local moment. In a sufficiently low-temperature regime, *E*_loc_(*δ*) separates the Kondo-screened regime with a large FS (FS_L_, 4*f*-electrons delocalized, right side of the *E*_loc_(*δ*) line) and the Kondo-destruction regime with a small FS (FS_S_, 4*f*-electrons localized, left side of the *E*_loc_(*δ*) line). The ground state is divided into three phases: an AFM phase with a small FS (AFM_S_), an AFM phase with a large FS (AFM_L_), and a paramagnetic phase with a large FS (P_L_). **a** For the conventional SDW type QCP, *E*_loc_(*δ*) terminates inside the AFM regime. **b** For a Kondo-breakdown type QCP, *E*_loc_(*δ*) terminates at the AFM QCP. Top and bottom insets show cartoon pictures of the FS and the spin fluctuations in the different phases. Olive, orange, and red arrows indicate the local moments, the itinerant conduction electrons, and the magnetic moments screened by the conduction electrons, respectively.

The Kondo singlet is a quantum state in which spins of surrounding conduction electrons collectively screen a local moment through their antiferromagnetic (AFM) exchange. Theoretically expected and experimentally confirmed^1,2^, the cloud of screening conduction electrons around a Kondo impurity extends radially to a distance *ξ* = ℏ*v*_F_/*k*_B_*T*_K_, where *v*_F_ is the conduction electron Fermi velocity and *k*_B_*T*_K_ is the energy scale of singlet formation. *ξ* can be up to micrometers and certainly greater than interatomic spacing. In the many-body process of Kondo-singlet formation, the spin of the local moment becomes part of the conduction electron Fermi volume. For a periodic lattice of Kondo impurities, typified by *f*-electron heavy-fermion metals, quantum coherence among Kondo singlets (qualitatively, a Bloch state of Kondo-screening clouds) results in the formation of highly entangled composite heavy quasiparticles as *T* → 0 K and an increase in the Fermi volume that counts both the local moments and conduction electrons. Interactions within the narrow (of order meV) quasiparticle bands can lead to an instability, often a spin-density-wave (SDW), of the large Fermi volume. Tuning the SDW transition to *T* = 0 K by a non-thermal control parameter, such as pressure, magnetic field, or chemical substitution, allows access to a quantum critical point (QCP) in which quantum fluctuations of the SDW order parameter control physical properties to temperatures well above *T* = 0 K^3–5^. The transition from a SDW order to a paramagnetic state at *T* = 0 K has no effect on the Fermi volume. This picture of a SDW QCP ignores the role of a long-range Rudermann-Kittel-Kasuya-Yosida (RKKY) interaction *I* among local moments that is mediated by the same conduction electrons that produce a Kondo singlet. The RKKY interaction induces dynamical correlations among the local moments, inhibiting Kondo singlet formation and thus preventing the emergence of a large Fermi volume. The competition between Kondo and RKKY interactions can be characterized by a non-thermal tuning parameter *δ* = *k*_B_*T*_K_/*I* ^6,7^. Relative to the Kondo scale, *I*, though always finite, is relatively insensitive to pressure, field, etc. so that the *T*–*δ* phase diagram illustrated in Fig. 1 for heavy-fermion systems is controlled primarily by changes in *T*_K_.

The nature of quantum critical fluctuations at *δ*_c_ where the magnetic boundary *T*_N_(*δ*) reaches *T* = 0 K changes qualitatively depending on the location of a crossover scale *E*_loc_(*δ*) that separates an electronic state in which static Kondo entanglement breaks down because of RKKY interactions and the Fermi surface is small (FS_S_) and a state with fully intact composite quasiparticles that produce a large Fermi surface (FS_L_). Depending on the position of *E*_loc_(*δ*), the nature of the magnetic QCP at *δ*_c_ is either of the SDW or Kondo-breakdown type. In the SDW scenario (Fig. 1a), *E*_loc_ remains finite at the QCP and terminates inside the ordered phase; only magnetic degrees-of-freedom are quantum critical at *δ*_c_^3^. In a Kondo-breakdown scenario (Fig. 1b), however, *E*_loc_ reaches zero temperature at the magnetic QCP and the concomitant reconstruction of the FS from small-to-large coincides with the onset of magnetic order, thus incorporating both charge and magnetic quantum fluctuations at the Kondo-breakdown QCP^8–11^. The distinct difference between SDW and Kondo-breakdown criticality has fundamental consequences for interpreting the origin of new phases of matter that frequently emerge around a QCP as the system relieves the buildup of entropy.

Evidence for a Kondo-breakdown type of quantum criticality has been found in a few heavy-fermion materials, notably CeCu_6-*x*_Au_*x*_^12^, YbRh_2_Si_2_^13,14^, and CeRhIn_5_;^15,16^ whereas, a phase diagram like that in Fig. 1a appears in CeIn_3_^17^, Co-doped YbRh_2_Si_2_^18^ and Ir-doped CeRhIn_5_^19,20^. Among examples representative of Fig. 1b physics, a FS change at their QCP has been inferred from Hall measurements. In CeRhIn_5_, on the other hand, evidence for an abrupt change in FS as *T* → 0 K has come from a pressure-dependent quantum oscillation study that directly probes the FS of CeRhIn_5_ in which an abrupt reconstruction of the FS coincides with an AFM QCP^15,21,22^. Evidence for the crossover scale *E*_loc_(*δ*), characteristic of Kondo breakdown, has yet to be reported in CeRhIn_5_, opening the possibility that FS reconstruction could be a consequence of a change in FS topology resulting from the loss of magnetic order^23^. Here, we probe the change in Hall effect via systematic control of external pressure to identify *E*_loc_(*δ*) in CeRhIn_5_, thereby demonstrating clearly that its criticality is of the Kondo-breakdown type. Replacing 4.4% of the In atoms by Sn shifts *E*_loc_(*δ*) such that it intersects the *T*_N_(*P*) boundary at finite temperature (Fig. 1a) and the magnetic criticality changes to the SDW type. Not only imposing a far more unambiguous interpretation of criticality in CeRhIn_5_, these discoveries point to a change in the nature of fluctuations leading to Cooper pairing in pure and Sn-doped CeRhIn_5_ and to the importance of *E*_loc_ for confirming a theoretically proposed beyond-Landau framework to understand quantum criticality^4,6^.

## Results

### Observation of *E*_loc_ in CeRhIn_5_

**Fig. 2:**
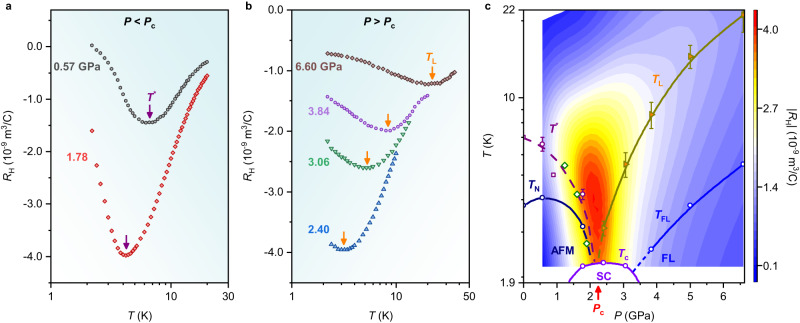
Evolution of the Hall coefficient with pressure and the *T*–*P* phase diagram for CeRhIn_5_. **a**, **b** Temperature dependence of Hall coefficient *R*_H_ for CeRhIn_5_ at representative pressures measured at temperatures above the superconducting transition temperature *T*_c_ and under a magnetic field of 1 T applied along the *c*-axis. The field is much lower than those required to observe quantum oscillations^15^ and to suppress superconductivity^21^. The purple and orange arrows represent the onset of short-range AFM spin correlations at T^*^ and the 4*f*-electron delocalization crossover temperature *T*_L_, respectively (see text for details). **c** *T*–*P* phase diagram of CeRhIn_5_ at 0 T overlaid with a contour plot of the amplitude of the Hall coefficient |*R*_H_| at 1 T. T^*^, *T*_N_, *T*_L_, *T*_c_, and *T*_FL_ are denoted by purple circles, navy circles, orange triangles, violet circles, and blue circles, respectively. Kondo breakdown and the AFM QCP coincide at the critical pressure *P*_c_ where *T*_c_ reaches a maximum, the Fermi surface reconstructs from small-to-large, and temperature *T*_L_ of the local extremum in |*R*_H_| extrapolates to zero temperature. The purple squares and olive diamonds are data adopted from Hall^24^ and nuclear quadrupole resonance^26^ measurements, respectively. The dashed and solid lines are guides to the eyes. AFM, SC, and FL stand for antiferromagnetic, superconducting, and Fermi liquid regions, respectively. Error bars on the T^*^ and *T*_L_ represent the uncertainties in determining the minimum in the Hall coefficient.

Previous Hall measurements of CeRhIn_5_ were limited to 2.6 GPa close to the critical pressure, *P*_c_ = 2.3 GPa, at which the AFM transition *T*_N_ extrapolates to *T* = 0 K and the superconducting transition temperature *T*_c_ reaches a maximum^21,22,24^. Our measurements of the Hall coefficient *R*_H_ at *P* < *P*_c_, Fig. 2a, are consistent with the primary features reported earlier^24^. A local minimum in *R*_H_ at T^*^ (as indicated by purple arrows) signals the onset of short-range AFM spin correlations above *T*_N_^25,26^ and is suppressed together with *T*_N_ under pressure. Figure 2b shows *R*_H_ in the high-pressure regime (*P* > *P*_c_) where a previously unidentified local minimum in *R*_H_ appears at *T*_L_ (as marked by the orange arrows) and moves to higher temperature as the system is tuned away from magnetic order with increasing pressure. (*T*_L_ appears well above the temperature below which the resistivity assumes a Fermi-liquid *T*^2^ dependence^16^.) The Hall coefficient in a multiband system with magnetic ions, particularly a compensated heavy-fermion metal like CeRhIn_5_, is not straightforward to interpret^15,24,27^. Prior experiments rule out any significant asymmetric (skew) scattering in CeRhIn_5_ at low temperatures and pressures below *P*_c_^24,27^, leaving temperature- and pressure-dependent changes in the ordinary Hall contribution as the most likely origin of *T*_L_. In a system like CeRhIn_5_, the magnitude of the ordinary term can be influenced not only by changes in carrier density but also by scattering rates on different parts of the Fermi surface, details of the surface topology, and the presence of spin fluctuations. Likely, all contribute to some extent to the unusual temperature- and pressure-dependence of an ordinary Hall contribution at *P* > *P*_c_ and to a change in the character of charge carriers at *T*_L_.

A color-contour map of the absolute value of *R*_H_ (|*R*_H_|) for CeRhIn_5_ is displayed in the temperature–pressure (*T*–*P*) plane in Fig. 2c. In the low-pressure AFM regime, T^*^ and *T*_N_ smoothly extrapolate to *T* = 0 K at *P*_c_, at which *T*_c_ and |*R*_H_| are a maximum. The existence of an AFM QCP at *P*_c_, hidden by the pressure-induced superconducting dome, has been revealed explicitly by applying a magnetic field sufficient to suppress superconductivity^21,22^. As shown in the figure, *T*_L_(*P*) extrapolates smoothly on the paramagnetic side of the diagram to *T* = 0 K at the magnetic QCP where, in the limit *T* → 0 K, the FS reconstructs, de Haas-van Alphen (dHvA) frequencies increase, and the quasiparticle effective mass diverges^15^. These are all essential characteristics of a Kondo-breakdown QCP. The phase diagram in Fig. 2c coincides with the theoretically predicted phase diagram (Fig. 1b) if we identify *T*_L_(*P*) as *E*_loc_(*P*), i.e., a change in the nature of charge carriers at *T*_L_ is accompanied by a crossover from small-to-large Fermi surface with increasing pressure at finite temperature. Theoretically, *E*_loc_ appears at some temperature below the onset of heavy quasiparticle formation^6^, typically taken experimentally to be signaled by the temperature *T*_max_ where the magnetic resistivity reaches a maximum. In CeRhIn_5_, *T*_max_(*P*) is roughly 6.5 times *T*_L_(*P*) at *P* > *P*_c_^28^. Multiple experiments, including dHvA measurements^15^, suggest that CeRhIn_5_ at *P* = *P*_c_ is equivalent to the isostructural heavy-fermion superconductor CeCoIn_5_ at *P* = 0 GPa^29^. Recent Hall measurements argue that CeCoIn_5_ at *P* = 0 GPa is very close to a QCP characterized by a localization/delocalization of 4*f*-electrons at a transition connecting two Fermi surfaces of different volumes^30^. A minimum in its Hall coefficient evolves with pressure increasing from *P* = 0 GPa in very much the same way as *T*_L_(*P*) shown in Fig. 2b for CeRhIn_5_ at *P* > *P*_c_^24^. Further, the pressure-dependent temperature of a Hall minimum tracks the delocalization crossover temperature determined by nuclear quadrupole resonance measurements in CeIn_3_^17,31^, providing additional support for associating the Hall minimum in CeRhIn_5_ and CeCoIn_5_ with a delocalization crossover at finite temperature. Finally, similar to T^*27^, the influence of magnetic field on *T*_L_ (Supplementary Fig. 1) is negligible even though |*R*_H_| is suppressed, an observation arguing against a substantial contribution of magnetic fluctuations to determining *T*_L_. Each of these further compels the identification of *T*_L_ with *E*_loc_.

### Quantum criticality in Sn-doped CeRhIn_5_

**Fig. 3:**
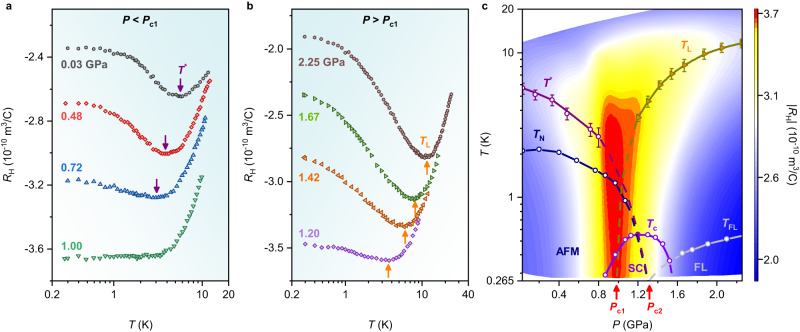
Evolution of the Hall coefficient with pressure and the *T*–*P* phase diagram for Sn-doped CeRhIn_5_. **a**, **b** Hall coefficient *R*_H_ of Sn-doped CeRhIn_5_ measured at 5 T is plotted as a function of temperature at representative pressures. The purple and orange arrows represent the onset of short-range magnetic correlations at T^*^ and the 4*f*-electron delocalization crossover temperature *T*_L_, respectively (see text for details). **c** *T*–*P* phase diagram of Sn-doped CeRhIn_5_ at 0 T overlaid with a contour plot of the amplitude of the Hall coefficient |*R*_H_| at 5 T. T^*^, *T*_N_, *T*_L_, *T*_c_, and *T*_FL_ are denoted by purple circles, navy circles, orange triangles, violet circles, and gray circles, respectively. *P*_c2_ (~1.3 GPa) is the critical pressure where *T*_N_ extrapolates to zero temperature, corresponding to an SDW QCP, and *T*_c_ reaches a maximum. *P*_c1_ (~1.0 GPa) is another critical pressure where *T*_L_(*P*) extrapolates to zero temperature, indicating that destruction of the Kondo effect occurs within the AFM phase. The dashed and solid lines are guides to the eyes. AFM, SC, and FL stand for antiferromagnetic, superconducting, and Fermi liquid regions, respectively. Error bars on the T^*^ and *T*_L_ represent the uncertainties in determining the minimum in the Hall coefficient.

We turn to the case of CeRhIn_5_ with a Sn concentration of 0.044, CeRh(In_0.956_Sn_0.044_)_5_, labelled as Sn-doped CeRhIn_5_ in the following. Figure 3a, b shows the temperature dependence of the Hall coefficient *R*_H_ for Sn-doped CeRhIn_5_ at representative pressures up to 2.25 GPa. *R*_H_ was obtained by applying a magnetic field of 5 T that completely suppresses superconductivity over the whole pressure range studied. Similar to pure CeRhIn_5_, two characteristic temperatures T^*^ and *T*_L_ are revealed by a local minimum in *R*_H_. In the low-pressure regime, T^*^ decreases monotonically with increasing pressure and becomes unresolvable at 1.0 GPa (Fig. 3a). At pressures higher than 1.0 GPa, another local minimum in *R*_H_ appears at *T*_L_ and increases with increasing pressure (Fig. 3b). We note that the field effects on T^*^ and *T*_L_ in Sn-doped CeRhIn_5_ are negligible (Supplementary Fig. 3). A color-contour map of the amplitude of *R*_H_ ( | *R*_H_ | ) for Sn-doped CeRhIn_5_ at 5 T is shown in the *T*–*P* plane in Fig. 3c, which is overlaid with the phase boundaries. The slight Sn doping leads to a decrease of *T*_N_ from 3.8 K for pure CeRhIn_5_ to 2.1 K in the Sn-doped case at ambient pressure^32^. With applying pressure, *T*_N_ decreases gradually and extrapolates to a terminal critical pressure *P*_c2_ (~1.3 GPa) where pressure-induced superconductivity reaches a maximum *T*_c_. Accompanying the suppression of *T*_N_, T^*^(*P*) decreases with pressure and also extrapolates to *T* = 0 K at *P*_c2_, a response qualitatively similar to pure CeRhIn_5_ in which T^*^(*P*) is associated with the development of short-range AFM correlations. With *T*_L_(*P*) « *T*_max_(*P*) (Supplementary Fig. 5 and ∂*T*_L_/∂*P* > 0 at *P* ≥ 1.2 GPa (Fig. 3b), as in CeRhIn_5_ at pressures above *P*_c_, we associate *T*_L_(*P*) with *E*_loc_(*P*) in Sn-doped CeRhIn_5_. In contrast to CeRhIn_5_, *E*_loc_(*P*) extrapolates to *T* = 0 K at a distinctly lower pressure *P*_c1_ (~1.0 GPa) than the critical pressure *P*_c2_ where AFM transition is suppressed to *T* = 0 K. The termination of *E*_loc_ at *P*_c1_ indicates that a local-to-itinerant transformation of 4 *f* degrees-of-freedom and concomitant reconstruction of FS take place within the AFM state of the Sn-doped material.

**Fig. 4:**
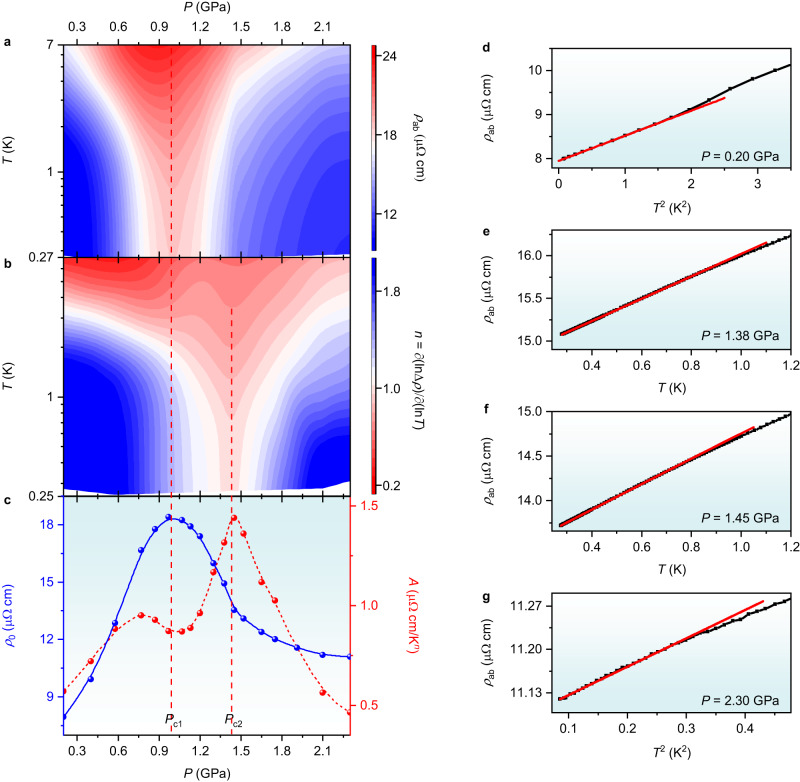
Quantum criticality of Sn-doped CeRhIn_5_ under pressure. **a** Contour plot of resistivity *ρ*_ab_ for Sn-doped CeRhIn_5_ measured parallel to the Ce-In plane under a magnetic field of 4.9 T. The significant enhancement of *ρ*_ab_ is centered around *P*_c1_. **b** Colours represent the local temperature exponent *n* derived from *n* = ∂(lnΔ*ρ*)/∂(ln*T*), where Δ*ρ* = *ρ*_ab_ – *ρ*_0_ = *AT*^*n*^ and *ρ*_0_ is the residual resistivity. A funnel regime with linear-*T* dependence of *ρ*_ab_ is observed around *P*_c2_, a characteristic of the non-Fermi liquid behavior near the AFM QCP, as shown in **e**, **f** at representative pressures of 1.38 and 1.45 GPa, respectively. **c** Pressure dependence of the residual resistivity *ρ*_0_ (left-axis, blue circles) and the coefficient *A* (right-axis, red circles) determined by fitting the low-temperature resistivity to *ρ*_ab_ = *ρ*_0_ + *AT*^*n*^. **d**–**g** show fits to representative data from which **c** is constructed. The dashed red lines in **a**–**c** are guides to the eyes. The red lines in **d**–**g** are least-squares fits to the low-temperature data.

The existence of two critical pressures in Sn-doped CeRhIn_5_ is supported by the low-temperature resistivity *ρ*_ab_ measured parallel to the Ce-In plane under a magnetic field of 4.9 T (Supplementary Fig. 6). The color contour of isothermal resistivity in the *T*–*P* plane, illustrated in Fig. 4a, shows a funnel of enhanced scattering centered at *P*_c1_. The local temperature exponent *n* derived from *n* = ∂(lnΔ*ρ*)/∂(ln*T*), in contrast, reveals a funnel of non-Fermi-liquid behavior centered near *P*_c2_ where the resistivity exhibits a linear-in-*T* dependence, illustrated in Fig. 4b. Figure 4d, g shows *ρ*_ab_ as a function of temperature at representative pressures of 0.20, 1.38, 1.45, and 2.30 GPa, respectively. A Landau-Fermi-liquid *T*^2^ dependence is observed at low- and high-pressure regimes in Fig. 4d, g, but the linear-*T* dependence is prominent near *P*_c2_ in Fig. 4e, f. Figure 4c summarizes the dependence on pressure of the residual resistivity *ρ*_0_ on the left ordinate and the temperature coefficient *A* on the right ordinate estimated from a fit to *ρ*_ab_ = *ρ*_0_ + *AT*^*n*^. With increasing pressure, *ρ*_0_ increases by over a factor of two, reaches a maximum at 1.0 GPa (=*P*_c1_), and decreases with increasing pressure, reflecting enhanced scattering of critical charge fluctuations around *P*_c1_^8–11^. The coefficient *A*, which is related to the effective mass of quasiparticles^19,33^, however, gradually increases at low pressures, goes through a local minimum at *P*_c1_, and peaks sharply at *P*_c2_, which is consistent with the divergence of effective mass predicted at an SDW QCP.

## Discussion

Despite several quantum-critical heavy-fermion candidates, the delocalization energy scale *E*_loc_ has been identified in a limited number of compounds, including YbRh_2_Si_2_^13,14,18^, Ce_3_Pd_20_Si_6_^34^, and CeIn_3_^17,31^. In the case of YbRh_2_Si_2_, *E*_loc_ manifests as anomalies in isothermal measurements, such as a step-like crossover in the field-dependent Hall coefficient and magnetoresistivity that sharpens with decreasing temperature or a smeared kink in the field-dependent Hall resistivity, magnetostriction, and magnetization^13,14,18^. The locus of *E*_loc_ points extrapolates to a field-tuned zero-temperature boundary of magnetic order, as in CeRhIn_5_ under applied pressure. Similar analysis of Hall data for Ce_3_Pd_20_Si_6_ finds, however, that *E*_loc_ extrapolates inside the ordered part of its field-dependent phase diagram^34^, analogous to Sn-doped CeRhIn_5_ (Fig. 3c). This also is the case with pressure-tuned CeIn_3_, discussed earlier, where a localization/delocalization scale intersects its antiferromagnetic phase at finite temperature^17,31^.

**Fig. 5:**
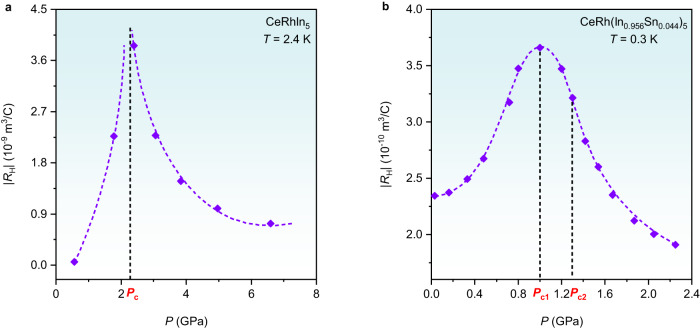
Isothermal pressure dependence of the Hall coefficient. **a** Pressure dependence of Hall coefficient |*R*_H_(*P*)| for CeRhIn_5_ at 2.4 K. *P*_c_ indicates the magnetic QCP. **b** Pressure dependence of Hall coefficient |*R*_H_(*P*)| for CeRh(In_0.956_Sn_0.044_)_5_ at 0.3 K. *P*_c2_ denotes the magnetic QCP. *P*_c1_ indicates the critical pressure where *T*_L_ extrapolates to zero temperature and is lower than the magnetic QCP *P*_c2_.

In these other examples, the signature for *E*_loc_ in various physical quantities has been used to infer a Fermi-surface change, but dHvA measurements unambiguously establish a jump in dHvA frequencies at the critical pressure where *T*_L_ extrapolates to *T* = 0 K in CeRhIn_5_. A simple interpretation of *R*_H_ would anticipate an associated step-like jump in carrier density at *P*_c_, but as shown in Fig. 5a, instead of having a sharp jump, the pressure-dependent isothermal Hall coefficient peaks strongly at *P*_c_. Perhaps measurements have not been made at sufficiently low temperatures to reveal a jump. More likely it is obscured by effects of critical charge and spin fluctuations on the Hall resistivity and/or only a small net change in the sum of hole and electron contributions in this nearly compensated metal. Though present experiments cannot make a definitive distinction, they do reflect the approach to FS reconstruction detected in dHvA that is coincident with a magnetic QCP. In contrast to the sharp peak in |*R*_H_(*P*)| in CeRhIn_5_, there is a broad maximum |*R*_H_(*P*)| that peaks at *P*_c1_ but encompasses both *P*_c1_ and *P*_c2_ in CeRh(In_0.956_Sn_0.044_)_5_ (Fig. 5b). Such a broad maximum relative to that in CeRhIn_5_ is not surprising because of the close proximity of two critical pressures, each with their own spectrum of critical spin/charge fluctuations, and smearing these spectra by disorder inherent to the Sn substitution.

The distinctly different critical behaviors of pure and Sn-doped CeRhIn_5_ are captured in a generalized quantum-critical phase diagram that includes both Kondo-breakdown and SDW criticality^8,35^. In this theory, the nature of quantum criticality is determined by two quantities, *δ* and *G* (Supplementary Fig. 9), where, as before, *δ* = *k*_B_*T*_K_/*I*, and *G* is a parameter that reflects magnetic frustration or effective spatial dimensionality. Our results are consistent with Kondo breakdown coinciding in pure CeRhIn_5_ under pressure with a *T* = 0 K transition from an AFM state with a small FS (AFM_S_) to a paramagnetic state with a large FS (*P*_L_) at *P*_c_, i.e., Kondo breakdown and the AFM QCP coincide. Similar to Sn-doped CeCoIn_5_^36^, Sn substitution for In in CeRhIn_5_ enhances hybridization between 4*f*- and conduction electron wave functions, i.e., increases *T*_K_ and thus *δ*. Stronger hybridization also reduces frustration among magnetic exchange pathways^8,37^. Analysis of magnetic neutron-diffraction experiments finds a clear change in magnetic structure and decrease of the ordered moment for a Sn concentration *x* ≥ 0.052, comparable to CeRh(In_0.956_Sn_0.044_)_5_ under a modest pressure_,_ that is attributed to an abrupt modification of the Fermi surface^38^. With these changes induced by Sn doping, the system follows a different trajectory that goes through an intermediate magnetically ordered state with a large FS (AFM_L_), i.e., Kondo breakdown occurs inside the AFM region, and thus the corresponding AFM QCP is of the SDW type as illustrated in Fig. 1a and Supplementary Fig. 9 and found in the prototypical Kondo-breakdown system YbRh_2_Si_2_ when Rh is replaced by a small amount of Co^18^.

Before identifying the crossover scale *E*_loc_ in pure and Sn-doped CeRhIn_5_, their criticality was ambiguous, possibly either Kondo-breakdown or SDW^32,39^. That ambiguity now is removed, with consequences for an interpretation of the origin of their pressure-induced superconductivity. At the SDW QCP in Sn-doped CeRhIn_5_, which is decoupled from the Kondo breakdown and where *T*_c_ reaches its maximum, quantum fluctuations of the magnetic order parameter are the prime candidate for mediating Cooper pairing^40^. Fluctuations around a Kondo-breakdown QCP, however, are far more complex and involve not only quantum-critical fluctuations of a magnetic order parameter but also of the Fermi surface, i.e., charge degrees-of-freedom^8–11^. Initial model calculations show that critical Kondo-breakdown fluctuations can produce a superconducting instability^41,42^, but much remains to make these calculations directly testable by experiment. Interestingly, *T*_c_ ≈ 2.3 K of CeRhIn_5_ at *P*_c_ and of CeCoIn_5_ at *P* = 0 GPa is among the highest of any rare-earth-based heavy-fermion superconductor.

The theory of Kondo-breakdown criticality in heavy-fermion materials has two essential signatures—an abrupt change from small-to-large Fermi surface coincident with magnetic criticality and the charge delocalization crossover scale *E*_loc_ that extrapolates from the paramagnetic state to the QCP^6^. Without both, Kondo-breakdown criticality cannot be established with certainty. Our Hall measurements provide compelling evidence for *E*_loc_ in pure and Sn-doped CeRhIn_5_ and, in light of dHvA results^15^, for Kondo-breakdown criticality in CeRhIn_5_. Finally, we speculate that critical charge fluctuations at a Kondo-breakdown QCP, evidenced by *ω*/*T* scaling of the frequency (*ω*)-dependent optical conductivity^10^, should play a non-trivial role in these signatures for *E*_loc_. Making this connection experimentally and theoretically would mark a significant advance.

## Methods

Single crystals of pure and Sn-doped CeRhIn_5_ were synthesized using the standard self-flux technique^32^. The high-pressure resistivity and Hall measurements on CeRhIn_5_ were carried out using the Van der Pauw method^43^ in a diamond-anvil cell made of Be-Cu alloy. NaCl powder was applied as the pressure medium to obtain a quasihydrostatic pressure environment. The pressure in the diamond-anvil cell was determined by the ruby fluorescence method^44^. The high-pressure resistivity and Hall measurements on CeRh(In_0.956_Sn_0.044_)_5_ were measured using the standard six-probe method in a Be-Cu/NiCrAl hybrid clamp-type cell. Daphne oil was employed as the pressure medium to obtain a hydrostatic pressure environment. The pressure dependence of the superconducting transition temperature of Pb was used to determine the pressure inside the clamp-type cell^45^. All measurements were performed with a low-frequency resistance bridge from Lake Shore Cryotronics. A ^4^He cryostat without magnetic field and a Physical Property Measurement System with a maximum magnetic field of 9 T were used in the temperature range of 1.8 to 300 K. A HelioxVL system with a maximum magnetic field of 12 T was used to control temperature down to 0.3 K.

### Supplementary information

Supplementary Information

Peer Review File

## Data Availability

All data supporting the findings of this study are available within the article and the supplementary information. The data are available upon request to the corresponding authors.
