# Supplementary material for: Evidence for charge delocalization crossover in the quantum critical superconductor CeRhIn5
Source: Nat Commun. 2023 Nov 13;14:7341. doi: 10.1038/s41467-023-42965-1 (PMC10643617; doi:10.1038/s41467-023-42965-1)
Supplement: Supplementary file 1 — Supplementary Information [file 41467_2023_42965_MOESM1_ESM.pdf]

Supplementary Information for

Evidence for charge delocalization crossover in the quantum critical superconductor $CeRhIn_5$

Honghong Wang[1,2,5], Tae Beom Park[1,2,3,5], Jihyun Kim[1,2], Harim Jang[1,2], Eric D. Bauer[4], Joe D. Thompson[4†], Tuson Park[1,2†]

[1]Center for Quantum Materials and Superconductivity (CQMS), Sungkyunkwan University, Suwon 16419, South Korea

[2]Department of Physics, Sungkyunkwan University, Suwon 16419, South Korea

[3]Institute of Basic Science, Sungkyunkwan University, Suwon 16419, South Korea

[4]Los Alamos National Laboratory, Los Alamos, NM 87545, USA

[5]These authors contributed equally: Honghong Wang, Tae Beom Park.

[†]email: jdt@lanl.gov; tp8701@skku.edu

The SI provides additional data and analysis that support the results in the main text.

Supplementary Note 1: Hall coefficient for $CeRhIn_5$ and $CeRh(In_{0.956}Sn_{0.044})_5$

Supplementary Note 2: Resistivity and phase diagram of $CeRh(In_{0.956}Sn_{0.044})_5$

Supplementary Note 3: Quantum critical trajectories

Supplementary Note 4: Superconductivity and pair breaking in $CeRh(In_{0.956}Sn_{0.044})_5$

Supplementary Figures

Supplementary References

Supplementary Note 1: Hall coefficient for $CeRhIn_5$ and $CeRh(In_{0.956}Sn_{0.044})_5$

Supplementary Figure 1 shows the Hall coefficient $R_H \equiv \rho_{xy}/B$ as a function of temperature for $CeRhIn_5$ at $P = 5.0$ GPa under various magnetic fields. The amplitude of R_H decreases under magnetic field, but the position of T_L is not affected by the application of different fields. Supplementary Figure 2 shows the Hall coefficient R_H as a function of temperature for $CeRh(In_{0.956}Sn_{0.044})_5$ at $P = 0.03$ GPa, which was measured under a magnetic field of 1 T applied along the *c*-axis (red symbols). As shown in the pure $CeMIn_5$ (M = Co, Rh, and Ir)[1], R_H follows the typical temperature dependence for heavy fermion metals above ~50 K. It is dominated by the anomalous Hall component due to intrinsic skew scattering that is proportional to $\chi\rho_{mag}$ (black symbols in Supplementary Fig. 2)[2]. Here, χ is the magnetic susceptibility, $\rho_{mag} = \rho - \rho_{ph} - \rho_0$ is the intrinsic magnetic contribution to resistivity, ρ_{ph} is the phonon contribution approximated by the non-magnetic analogue $LaRhIn_5$, and ρ_0 is the residual resistivity. Below 50 K, R_H shows a precipitous negative drop and reaches a local minimum at about 5.68 K (T^*, denoted by the purple arrow) before starting to increase with further decreasing temperature. This temperature variation of R_H is inconsistent with that of an anomalous Hall contribution, indicating that the low-temperature R_H is determined by the temperature dependent normal Hall contribution. Supplementary Figures 3a–c compare the amplitude of R_H under various magnetic fields at representative pressures of 0.03, 0.48, and 1.30 GPa, respectively. The amplitude of R_H slightly decreases under magnetic field in the low-pressure regime but is almost independent of the field at 1.30 GPa. Regardless of the pressure value, however, the position of T^* and T_L is not affected by the application of different fields. The weak field dependence of Hall coefficient in $CeRh(In_{0.956}Sn_{0.044})_5$ is further supported by the almost linear field dependence of Hall resistivity, as shown in Supplementary Fig. 4.

Supplementary Note 2: Resistivity and phase diagram of $CeRh(In_{0.956}Sn_{0.044})_5$

Supplementary Figures 5a,b display the temperature dependence of magnetic resistivity $\rho_{mag,ab}$ for $CeRh(In_{0.956}Sn_{0.044})_5$ at various pressures up to 2.30 GPa. A phonon contribution estimated from the resistivity of $LaRhIn_5$ has been subtracted from the measured resistivity to obtain $\rho_{mag,ab}$. The dependence on temperature of $\rho_{mag,ab}$ is typical of heavy fermion compounds[3-5]. For instance, at $P = 0.03$ GPa, $\rho_{mag,ab}$ exhibits a $-\ln T$ dependence with decreasing temperature from room temperature, which is characteristic of incoherent Kondo

scattering on the excited crystal electric field levels, and forms a broad maximum at T_1. Upon further cooling, $\rho_{\mathrm{mag,ab}}$ decreases and develops a small but discernible hump at T_2, characteristic of the onset of coherent Kondo scattering in the ground state. Another anomaly at lower temperatures is the inflection of $\rho_{\mathrm{mag,ab}}$ that is due to the reduction of spin-flip scattering with the formation of long-range AFM order below $T_N = 2.1$ K. With increasing pressure, pressure-induced superconductivity appears and reaches a maximum T_c of 0.55 K at 1.30 GPa, as shown in Supplementary Fig. 5c.

The T–P phase diagram of $CeRh(In_{0.956}Sn_{0.044})_5$ is displayed on top of a color-coded map of $\rho_{\mathrm{mag,ab}}$ in Supplementary Fig. 5d. T_1 decreases initially with applied pressure, whereas the feature at T_2 gradually increases and merges with T_1 at 0.87 GPa before increasing with further increasing pressure. This behavior is found in pure $CeRhIn_5$[4]. T_N is suppressed by pressure and becomes indiscernible above 1.07 GPa. It extrapolates to $T = 0$ K at the critical pressure P_{c2} (~1.3 GPa) where the dome of superconductivity is maximum, indicating a putative AFM QCP. $\rho_{\mathrm{mag,ab}}$ is strongly enhanced in the vicinity of a lower critical pressure P_{c1} (~1.0 GPa), showing a funnel-shape topology as evidenced by the orange color in the contour map.

To study quantum criticality in $CeRh(In_{0.956}Sn_{0.044})_5$ at lower temperatures, a field of 4.9 T is applied to suppress superconductivity completely in the studied pressure range (Supplementary Fig. 6a). $\rho_{\mathrm{mag,ab}}$ at low temperatures is strongly enhanced around P_{c1} (Supplementary Fig. 6b). Supplementary Figure 7 shows first derivative of resistivity at 0 T and 4.9 T for representative pressures above 0.77 GPa and the T–P phase diagram of $CeRh(In_{0.956}Sn_{0.044})_5$. The AFM transitions at 0 T (square symbols) and 4.9 T (cross symbols) overlap, indicating that T_N is almost independent of the magnetic field. At 1.2 GPa, which is higher than the critical pressure P_{c1} of 1.0 GPa, T_N is 0.73 K and T_L is 3.56 K. Observation of both T_N and T_L at 1.2 GPa shows that both characteristic temperatures remain finite in the critical regime between P_{c1} and P_{c2}, supporting that they are decoupled and terminate at two different critical pressures. Further power-law analysis of the low-temperature resistivity supports the existence of critical pressures at P_{c1} and P_{c2} as discussed in the main text. In addition, the low-temperature resistivity is also fitted by $\rho = \rho_0 + A^* T^2$ in the Fermi-liquid regime above P_{c2}, as shown in Supplementary Fig. 8a–c for representative pressures. The obtained pressure dependence of the coefficient A^* increases as pressure approaches P_{c2} from

the paramagnetic regime, as shown in Supplementary Fig. 8d, which is similar to the coefficient A obtained from the power-law fitting.

Supplementary Note 3: Quantum critical trajectories

Supplementary Figure 9 is a theoretical global phase diagram of quantum phase transitions that is generated as a function of $\delta = k_B T_K/I$ and quantum frustration (G)[6,7]. As a function of pressure, pure $CeRhIn_5$ follows trajectory II that goes through a direct transition from an AFM state with a small FS (AFM_S) to a paramagnetic state with a large FS (P_L), whereas Sn-doped $CeRhIn_5$ follows trajectory III that goes through an intermediate AFM state with a large FS (AFM_L) before transitioning to a paramagnetic state with a large FS (P_L).

Supplementary Note 4: Superconductivity and pair breaking in $CeRh(In_{0.956}Sn_{0.044})_5$

The residual resistivity ρ_0 reflects impurity scattering, which is 6.86 μΩ cm in $CeRh(In_{0.956}Sn_{0.044})_5$ at ambient pressure and about 343 times larger than that in pure $CeRhIn_5$ (~0.02 μΩ cm) due to the increase of impurity disorder induced by Sn substitution. Quantum critical fluctuations further enhance ρ_0[8] to 15.98 μΩ cm at the AFM QCP (~1.3 GPa) where T_c reaches its maximum of 0.55 K, which is 1.75 K lower than that of pure $CeRhIn_5$ (2.3 K) at its optimal pressure. The pair breaking rate can be quantified by the scattering rate caused by the nonmagnetic impurities, which, in our case, is Sn, *i.e.*, $1/\tau = (ne^2\Delta\rho)/m^* = \Delta\rho/\mu_0\lambda^2$, where $\Delta\rho$ (= $\rho(CeRh(In_{0.956}Sn_{0.044})_5) - \rho(CeRhIn_5)$) is 11.59 μΩ cm at the optimal pressure, μ_0 is the magnetic permeability of free space, and λ is the superconducting penetration depth that can be approximated roughly by its value in $CeRhIn_5$ at 2.26 GPa (λ = 338 nm)[9]. Thus, the maximum rate of the T_c suppression by pair breaking $dT_c/d(1/\tau)$ is –0.04, which is significantly weaker than that expected for d-wave superconductors based on Abrikosov-Gorkov theory, where $dT_c/d(1/\tau) = -\pi/4$[10]. Even if λ for Sn-doped $CeRhIn_5$ were overestimated by a factor of two, $dT_c/d(1/\tau)$ still would be far less than expected. Similar remarkable robustness of superconductivity to nonmagnetic impurities has been reported in $CeCoIn_5$[11]. It has been argued that odd-frequency p-wave spin-singlet pairing is robust against nonmagnetic impurities[12], raising the possibility that the pairing symmetry may have changed from the d-wave to p-wave spin-singlet pairing at the crossover between small and large FSs as pressure passes through P_{c1}[13]. A direct probe of the pair symmetry would test this possibility.

Supplementary Figures

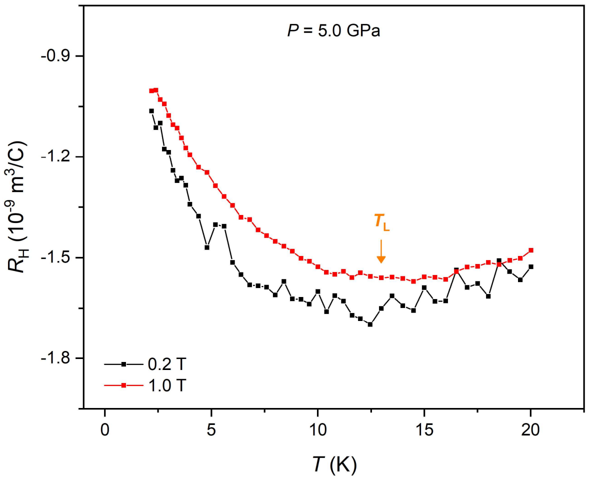

Supplementary Figure 1 | Hall coefficient for $CeRhIn_5$. Temperature dependence of the Hall coefficient R_H for $CeRhIn_5$ at 5.0 GPa measured at different magnetic fields along the *c*-axis. The orange arrow represents the 4*f*-electron delocalization crossover temperature T_L.

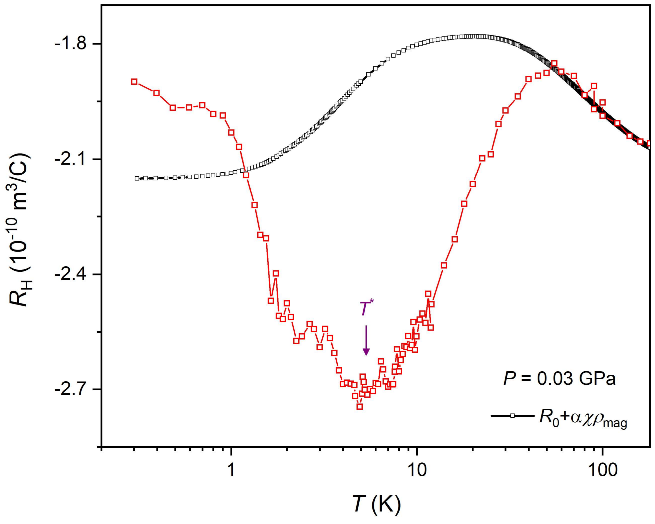

Supplementary Figure 2 | Hall coefficient for CeRh($In_{0.956}Sn_{0.044}$)$_5$. Temperature dependence of Hall coefficient R_H for CeRh($In_{0.956}Sn_{0.044}$)$_5$ at 0.03 GPa under a magnetic field of 1 T along the c-axis. The black symbols are the fit of high temperature R_H by $R_H = R_0 + R_a = R_0 + \alpha\chi\rho_{mag}$ and its extrapolation to lower temperatures on the basis of the independently measured $\chi(T)$ and $\rho_{mag}(T)$ and parameter α extracted from a fit to R_H at high temperature. R_0 is normal Hall coefficient contribution; R_a is anomalous Hall component due to the intrinsic skew scattering, $R_a = \alpha\chi\rho_{mag}$, where α is positive constant, χ is the magnetic susceptibility, and $\rho_{mag} = \rho - \rho_{ph} - \rho_0$ is the intrinsic magnetic contribution to resistivity. The phonon contribution ρ_{ph} is approximated by the non-magnetic analogue $LaRhIn_5$, and ρ_0 is the residual resistivity. The purple arrow indicates the local minimum of R_H, which reflects the development of short-range spin correlations at T^*.

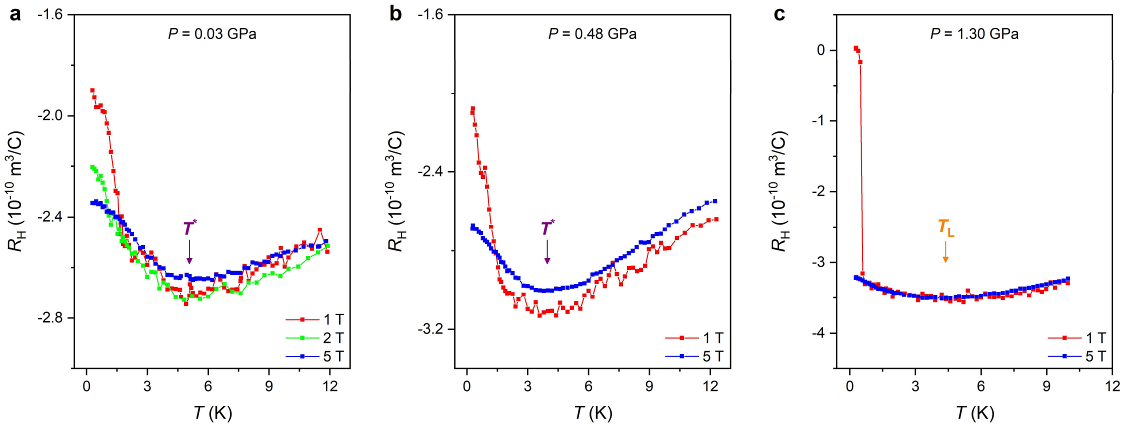

Supplementary Figure 3 | Hall coefficient for $CeRh(In_{0.956}Sn_{0.044})_5$ under different fields. **a–c,** Temperature dependence of the Hall coefficient R_H for $CeRh(In_{0.956}Sn_{0.044})_5$ at representative pressures measured at different magnetic fields along the *c*-axis. The purple and orange arrows represent the onset of short-range AFM spin correlations at T^* for pressures below P_{c1} and the 4*f*-electron delocalization crossover temperature T_L for pressures above P_{c1}, respectively.

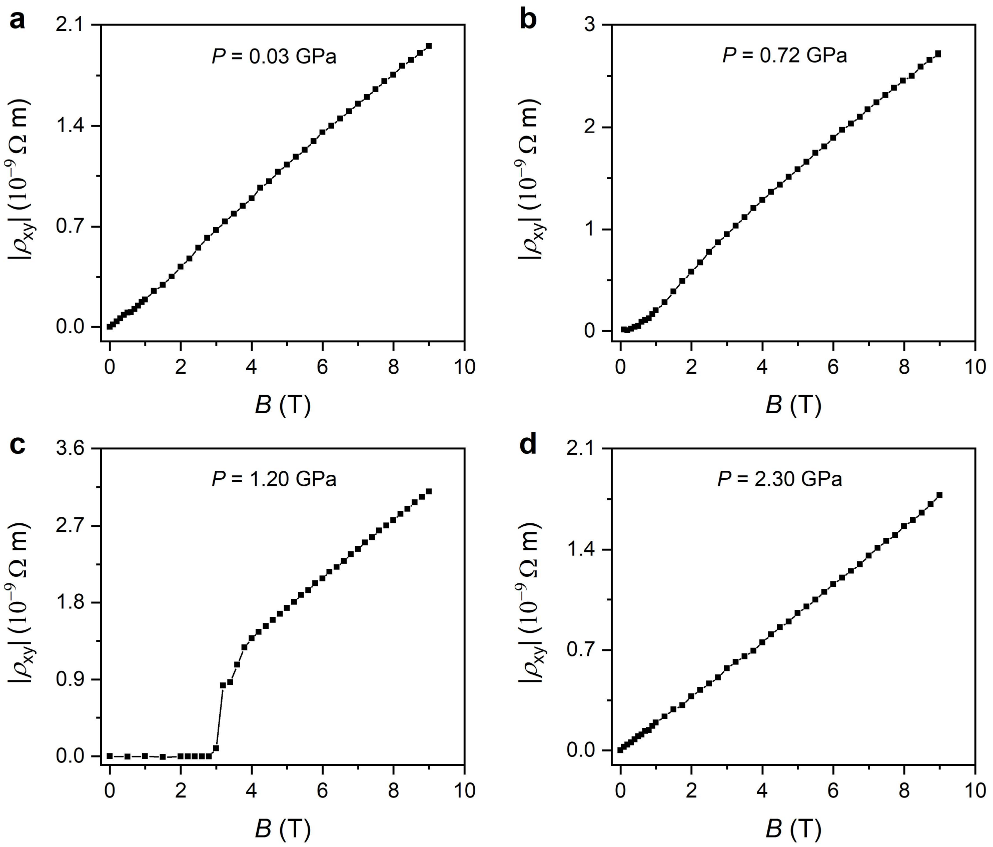

Supplementary Figure 4 | Weak field dependence of Hall coefficient in $CeRh(In_{0.956}Sn_{0.044})_5$. a–d, Field dependence of Hall resistivity for $CeRh(In_{0.956}Sn_{0.044})_5$ at representative pressures measured at 0.3 K.

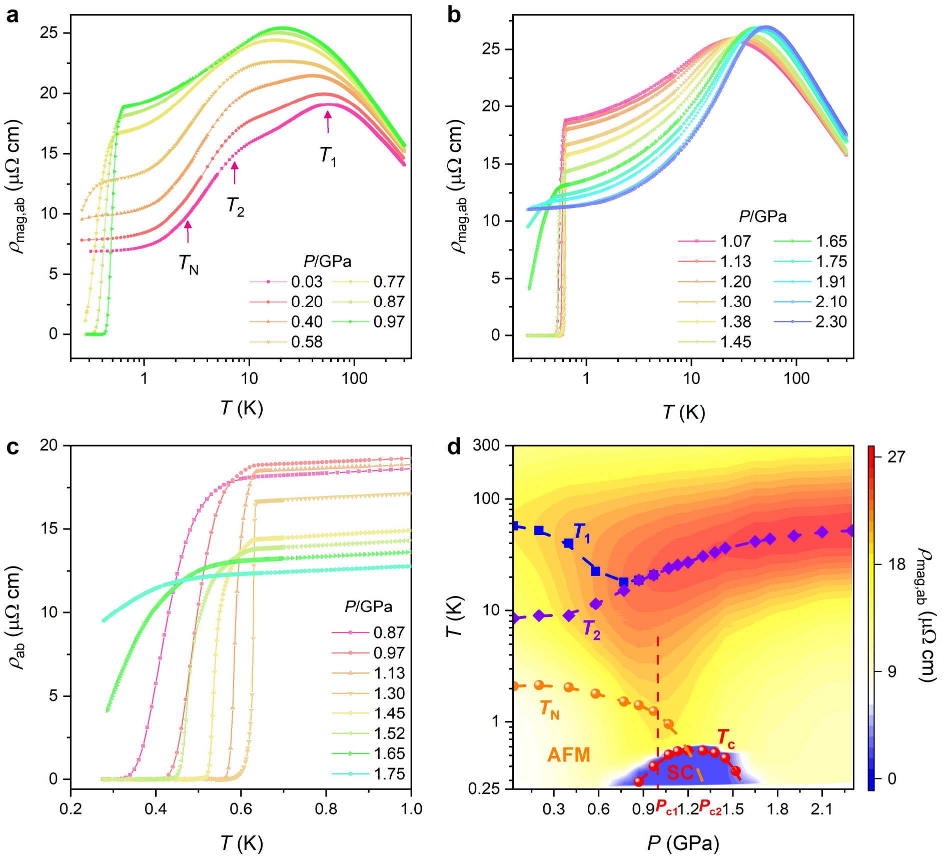

Supplementary Figure 5 | Resistivity and phase diagram of CeRh($In_{0.956}Sn_{0.044}$)$_5$ under pressure. a,b, Magnetic resistivity $\rho_{mag,ab}$ of CeRh($In_{0.956}Sn_{0.044}$)$_5$ is plotted as a function of temperature on a semi-logarithmic scale for $P \leq 0.97$ GPa and $P \geq 1.07$ GPa, respectively. The arrows in **(a)** mark the resistivity maximum (T_1), hump (T_2) and AFM transition (T_N) temperatures at P = 0.03 GPa. **c,** Electrical resistivity ρ_{ab} near the superconducting phase transition at various pressures. **d,** T–P phase diagram is overlaid with a color contour plot of $\rho_{mag,ab}$. T_1, T_2, and T_N are represented by blue squares, violet diamonds, and orange circles, respectively. The superconducting transition temperature T_c is denoted by red circles, which was determined by the zero-resistivity temperature. T_c reaches a maximum at the critical pressure P_{c2} (~1.3 GPa) where T_N extrapolates to T = 0 K, implying a putative AFM QCP. $\rho_{mag,ab}$ is strongly enhanced around a lower critical pressure P_{c1} (~1.0 GPa). The dashed lines are guides to the eyes. AFM and SC stand for antiferromagnetic and superconducting regions, respectively.

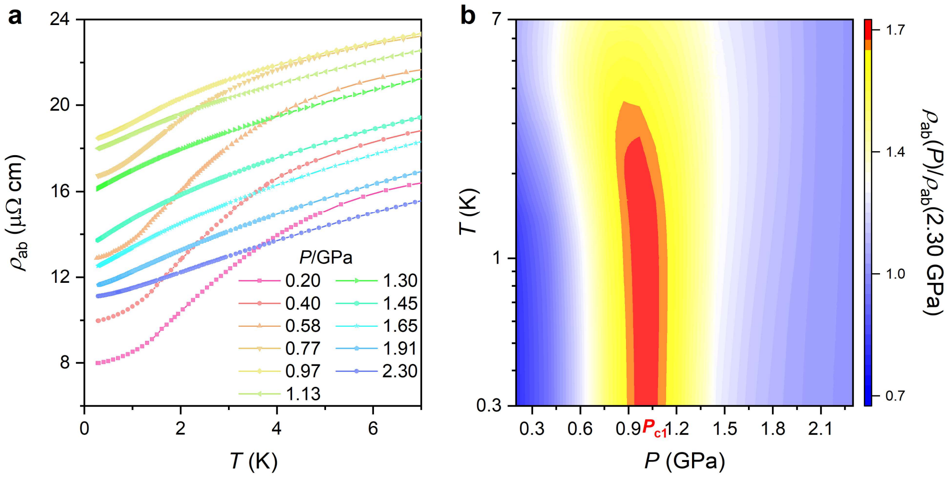

Supplementary Figure 6 | Resistivity in the tetragonal *ab*-plane of CeRh($In_{0.956}Sn_{0.044}$)$_5$ at 4.9 T. a, Temperature dependence of resistivity ρ_{ab} for CeRh($In_{0.956}Sn_{0.044}$)$_5$ at representative pressures up to 2.30 GPa. **b,** Contour plot of the resistivity normalized by its value at 2.30 GPa. The enhancement of resistivity is centered around P_{c1}.

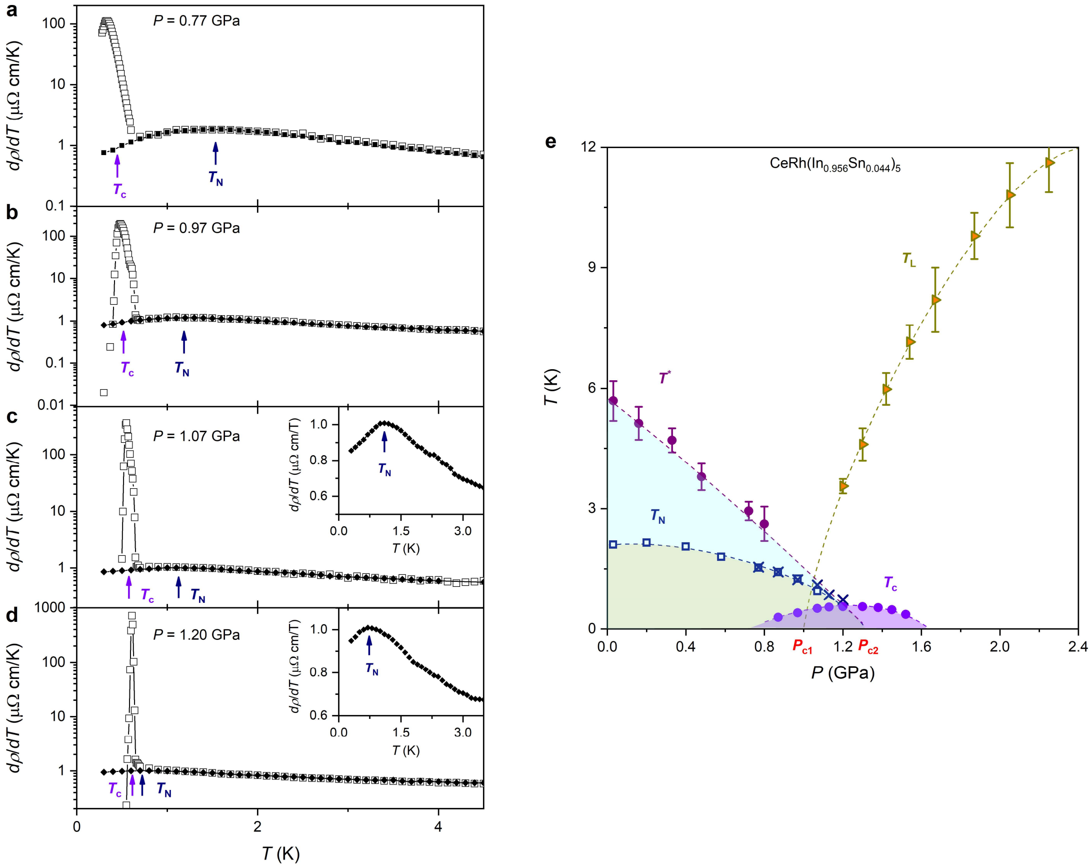

Supplementary Figure 7 | Two critical pressure points in CeRh($In_{0.956}Sn_{0.044}$)$_5$ at 4.9 T. a–d, The first derivative of resistivity at 0 T (open symbols) and 4.9 T (solid symbols) for representative pressures. Inserts of (**c**) and (**d**) show an enlarged view of the first derivative of resistivity at 4.9 T in the low-temperature regime. The violet and navy arrows indicate the superconducting and AFM transitions, respectively. **e,** *T*–*P* phase diagram of CeRh($In_{0.956}Sn_{0.044}$)$_5$. The navy squares and crosses represent the AFM transition determined from the first derivative of resistivity at 0 T and 4.9 T, respectively. The violet circles denote the superconducting transition temperature T_c determined by the zero-resistivity temperature. The purple circles and orange triangles represent the onset of short-range magnetic correlations at T^* and the 4*f*-electron delocalization crossover temperature T_L, obtained by the minimum of Hall coefficient $R_H(T)$ at 5 T. Error bars on the T^* and T_L represent the uncertainties in determining the minimum in the Hall coefficient.

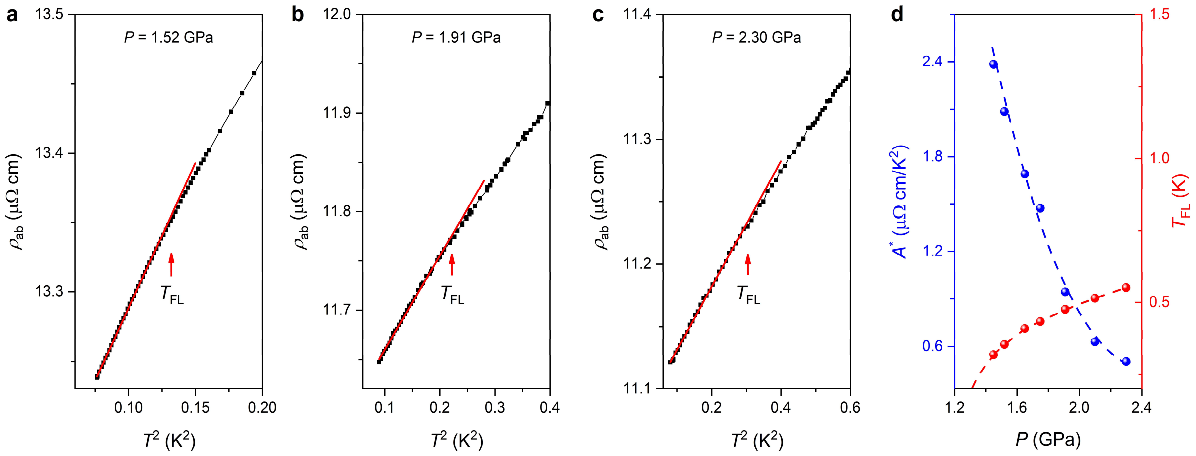

Supplementary Figure 8 | Fermi-liquid fitting of the resistivity of $CeRh(In_{0.956}Sn_{0.044})_5$ above the magnetic quantum critical pressure. a–c, Low-temperature resistivity is plotted against T^2 at representative pressures above P_{c2}. Red arrows mark the Fermi-liquid temperature T_{FL} below which $\rho = \rho_0 + A^*T^2$. **d**, Pressure dependence of the coefficient A^* (left-axis, blue circles) and the Fermi-liquid temperature T_{FL} (right-axis, red circles) determined by a deviation from a T^2 temperature dependence.

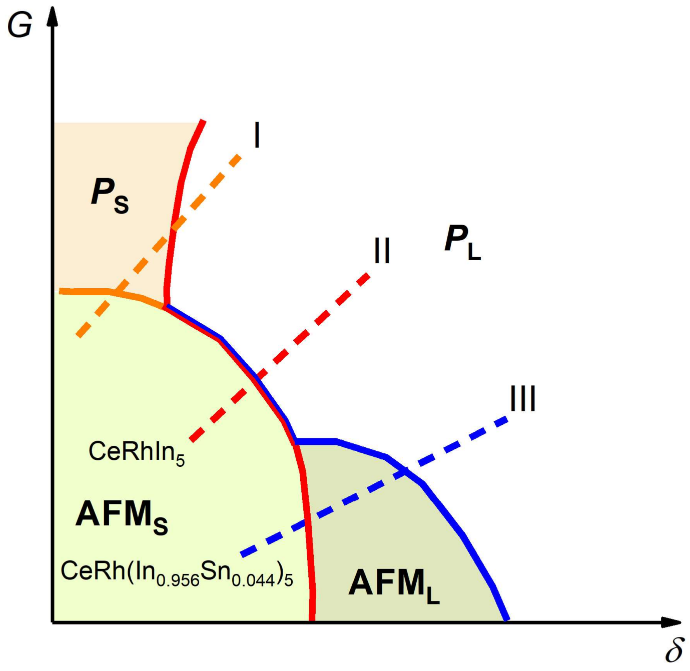

Supplementary Figure 9 | Global phase diagram for heavy fermion metals at zero temperature. δ is the ratio of Kondo and RKKY interactions $k_B T_K/I$, and G reflects a measure of frustration that is imposed, for example, by the crystal lattice, magnetic exchange or reduced spatial dimensionality. The red and blue (orange) lines represent the Kondo breakdown and the AFM transition, respectively. AFM_S, AFM_L, P_S, and P_L denote the AFM phase with small FS, the AFM phase with large FS, the paramagnetic phase with small FS, and the paramagnetic phase with large FS, respectively. Three types of trajectories are described by the lines "I", "II", and "III". The expected trajectories of $CeRhIn_5$ and $CeRh(In_{0.956}Sn_{0.044})_5$ are consistent with paths "II" and "III" in the phase diagram, respectively.

Supplementary References

1. Hundley, M. F., Malinowski, A., Pagliuso, P. G., Sarrao, J. L. & Thompson, J. D. Anomalous *f*-electron Hall effect in the heavy-fermion system Ce*T*In_5 (*T*=Co, Ir, or Rh). *Phys. Rev. B* **70**, 035113 (2004).
2. Fert, A. & Levy, P. M. Theory of the Hall effect in heavy-fermion compounds. *Phys. Rev. B* **36**, 1907 (1987).
3. Ren, Z. et al. Giant overlap between the magnetic and superconducting phases of $CeAu_2Si_2$ under pressure. *Phys. Rev. X* **4**, 031055 (2014).
4. Ren, Z. et al. Coincidence of magnetic and valence quantum critical points in $CeRhIn_5$ under pressure. *Phys. Rev. B* **96**, 184524 (2017).
5. Seyfarth, G. et al. Heavy fermion superconductor $CeCu_2Si_2$ under high pressure: multiprobing the valence crossover. *Phys. Rev. B* **85**, 205105 (2012).
6. Si, Q. Quantum criticality and global phase diagram of magnetic heavy fermions. *Phys. Status Solidi B* **247**, 476-484 (2010).
7. Coleman, P. & Nevidomskyy, A. H. Frustration and the Kondo effect in heavy fermion materials. *J. Low Temp. Phys.* **161**, 182-202 (2010).
8. Miyake, K. & Narikiyo, O. Enhanced impurity scattering due to quantum critical fluctuations: perturbational approach. *J. Phys. Soc. Jpn.* **71**, 867-871 (2002).
9. Heffner, R. H. et al. μSR study of $CeRhIn_5$ under applied pressure. *J.Phys.: Conf. Ser.* **225**, 012011 (2010).
10. Abrikosov, A. A. & Gor'kov, L. P. Contribution to the theory of superconducting alloys with paramagnetic impurities. *Sov. Phys. JETP* **12**, 1243-1253 (1961).
11. Gofryk, K. et al. Electronic tuning and uniform superconductivity in $CeCoIn_5$. *Phys. Rev. Lett.* **109**, 186402 (2012).
12. Yoshioka, Y. & Miyake, K. Impurity effect on frequency dependent superconductivity: odd-fequency piring and even-fequency piring. *J. Phys. Soc. Jpn.* **81**, 093702 (2012).
13. Otsuki, J. Competing *d*-wave and *p*-wave spin-singlet superconductivities in the two-dimensional Kondo lattice. *Phys. Rev. Lett.* **115**, 036404 (2015).
